# High chance that current atmospheric greenhouse concentrations commit to warmings greater than 1.5 °C over land

**DOI:** 10.1038/srep30294

**Published:** 2016-07-27

**Authors:** Chris Huntingford, Lina M. Mercado

**Affiliations:** 1 Centre for Ecology and Hydrology, Wallingford, Oxfordshire, OX10 8BB, UK; 2 College of Life and Environmental Sciences, University of Exeter, Amory Building, Rennes Drive, Exeter, EX4 4RJ, UK

## Abstract

The recent Paris UNFCCC climate meeting discussed the possibility of limiting global warming to 2 °C since pre-industrial times, or possibly even 1.5 °C, which would require major future emissions reductions. However, even if climate is stabilised at current atmospheric greenhouse gas (GHG) concentrations, those warming targets would almost certainly be surpassed in the context of mean temperature increases over land only. The reason for this is two-fold. First, current transient warming lags significantly below equilibrium or “committed” warming. Second, almost all climate models indicate warming rates over land are much higher than those for the oceans. We demonstrate this potential for high eventual temperatures over land, even for contemporary GHG levels, using a large set of climate models and for which climate sensitivities are known. Such additional land warming has implications for impacts on terrestrial ecosystems and human well-being. This suggests that even if massive and near-immediate emissions reductions occur such that atmospheric GHGs increase further by only small amounts, careful planning is needed by society to prepare for higher land temperatures in an eventual equilibrium climatic state.

The year 2015 United Nations meeting on climate change (Conference of Parties, COP21) re-iterated the desire to constrain global warming to within two degrees since the pre-industrial period, and additionally pursue efforts “to limit the temperature increase to 1.5 °C above pre-industrial levels”^1^. Even to achieve the two-degrees warming target will require year-on-year emissions reductions of at least 2% per annum or more, and starting soon^2^. Society has already used two thirds of the “allowable” cumulative carbon dioxide (CO_2_) emissions^3^ to stay below the two-degrees target, when accounting for effects of other radiatively-active greenhouse gases (GHGs) such as methane and nitrous oxide^4^. Making transitions from fossil fuel usage will pose major challenges to society, and so it is important to understand what will be gained and furthermore, whether even this will guarantee safe future climatic states.

Three issues affect discussion of atmospheric greenhouse gas concentrations levels to ensure safe upper limits of global warming for society. First oceans currently provide a significant thermal inertia of many decades^5^. Hence temperatures in a warming climate are lower than any eventual equilibrium stabilisation value for equivalent atmospheric GHG concentrations. Equilibrium temperatures are often referred to as “committed” warmings for any altered atmospheric concentrations. It is noted that if GHG emissions are simply stopped, then atmospheric temperatures will subsequently reduce very slowly^6^, suggesting it may be difficult to return rapidly from any committed warming levels that are found to be dangerous. Second, equilibrium climate sensitivity relating GHG concentrations to any final stable warming levels remains highly uncertain across the current generation of climate models^7^. Third, temperature increases over land are projected to be significantly higher than for oceans and hence the global mean. Hence even stabilisation at planetary i.e. global mean thresholds of 1.5 °C or 2.0 °C could correspond to higher warming levels being experienced by much of society. This land-ocean contrast was first noted in early climate modelling studies^8^,^9^, and the underlying processes have since been investigated in detail^10^,^11^. Here we sample across climate models to understand these three factors, and specifically in the context of estimating equilibrium warming over land associated with contemporary year 2015 greenhouse gas concentrations.

To calculate the land-ocean contrast that yields higher terrestrial warming amounts, we analyse diagnostics from Global Climate Models (GCMs) submitted to the Coupled Model Intercomparison Project Phase 5 (CMIP5)^12^. Models are considered for which the equilibrium climate sensitivity Δ*T*_2 × CO2_ (°C) (i.e. global equilibrium warming associated with a doubling of atmospheric CO_2_ concentrations) is presented in the 5^th^ Intergovernmental Panel on Climate Change (IPCC) report, Table 9.5^13^. For each model, we analyse annual mean air temperatures at 1.5 m above the land surface, and derive area-weighted averages for the globe, land and ocean. Models are only retained where temperature projections exist for both the scenario of altered atmospheric GHGs compatible with little future action to reduce emissions “rcp85”, and of major reductions with high mitigation (“rcp26” if available, otherwise “rcp45”)^14^. Changes in these quantities since pre-industrial times are calculated as anomalies from the mean of the first four decades of simulations, which generally start around year 1850. This gives global, land and ocean temperature changes named Δ*T*_Global_ (°C), Δ*T*_Land_ (°C) and Δ*T*_Ocean_ (°C) respectively. By definition, for fraction of the Earth’s surface as ocean, *f*, then Δ*T*_Global_ = *f*Δ*T*_Ocean_ + (1−*f* )Δ*T*_Land_.

## Results

**Figure 1:**
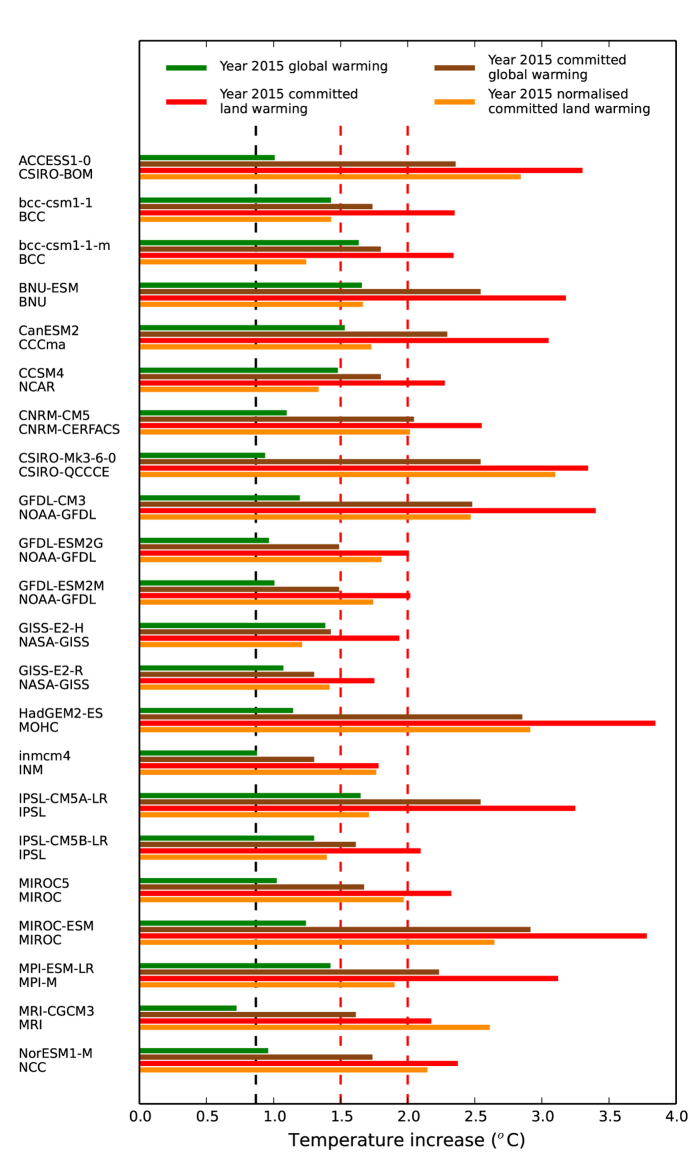
Warming levels for contemporary GHG concentrations. For each GCM and climate research centre named on the vertical axis, shown in green is global mean warming for rcp85 simulations, centred on year 2015. This is an ensemble average if multiple rcp85 simulations are available for any particular model. Associated global committed warming for year 2015 GHG concentrations are brown, utilising reported model climate sensitivities in IPCC 5^th^ report. Using these calculations, then presented in red is committed warming over land only, based on model estimates of land-sea temperature contrast. In orange are normalised land warmings, accounting for differences between each modelled and observed year 2015 global warming. The latter observed value is marked as the vertical dashed black line, and is from the NCEP climatology. Warming levels of 1.5 °C and 2.0 °C are marked as vertical red dashed lines.

In Fig. 1 we first present in green for each model the contemporary global warming projection, Δ*T*_Global, 2015_ (°C). This is under rcp85 scenario, and calculated as the time-average value over 15 years centred on year 2015. The emissions implied by the rcp85 concentration scenario are presently near to the actual levels of fossil fuel burning. Where more than one rcp85 simulation is available for a given GCM, we show the mean across those ensemble members for each climate model.

Radiative forcing Δ*Q* (W m^−2^) is a summary statistic of net change to the Earth’s overall energy balance due to atmospheric gas composition changes. The value of this corresponding to a doubling of atmospheric CO_2_ concentration satisfies^15^ Δ*Q*_2 × CO2_ = 5.35 × log(2.0) (W m^−2^), whilst the anthropogenic radiative forcing associated with rcp85 scenario in year 2015 and across all radiatively active gas changes since pre-industrial times is given as Δ*Q*_2015_ = 2.30 W m^−2 ^14. This allows a scaling by radiative forcing to derive, with knowledge of the climate sensitivity of each model^13^ Δ*T*_2 × CO2_, a set of GCM-dependent estimates of global committed temperature rise for current levels of atmospheric GHG changes. These we name as Δ*T*_Global, Commit, 2015_(°C), are shown as brown in Fig. 1 and thus calculated as Δ*T*_Global, Commit, 2015_ *=* (Δ*Q*_2015_/Δ*Q*_2 × CO2_) × Δ*T*_2 × CO2_. Many values of committed global temperature rise Δ*T*_Global, Commit, 2015_ are greater than 1.5 °C, and some models predict warming levels higher even than the 2.0 °C threshold.

The mean committed warming over land, Δ*T*_Land, Commit, 2015_ (°C), can then be finally calculated with knowledge of ocean fraction, *f*, committed global warming Δ*T*_Global, Commit, 2015_ and using the predicted land-sea warming contrast for each model, *υ* = Δ*T*_Land_/Δ*T*_Ocean_. This satisfies Δ*T*_Land, Commit, 2015_ = Δ*T*_Global, Commit, 2015_/[(*f*/*υ*) + 1−*f* ], and these values are given in red in Fig. 1. This shows that for current levels of atmospheric GHG concentrations, for all models the implied committed mean warming over land is greater than 1.5 °C and most additionally exceed 2.0 °C.

The vertical dashed black line of Fig. 1 is global warming increase since period 1880–1909 based on the NCDC/NESDIS/NOAA historical temperature records^16^, Δ*T*_Global, NCEP_(°C), and presented as mean of period 2001–2015. This value is significantly lower than almost all model projections (compare to green bars), although noting the small offset of 7 years as model predictions are 15 years centred on 2015. These higher model-predicted temperature changes have been the subject of debate and controversy. This is sometimes referred to as the climate change “Hiatus”, leading to investigation of possible causes related to natural variability e.g. ref. [17]. To provide an uncertainty range and to acknowledge this apparent discrepancy between climate models and observations for present day, we additionally normalise Δ*T*_Land, Commit, 2015_ by multiplication with factor (Δ*T*_Global, NCEP_/Δ*T*_Global, 2015_). These scaled values, Δ*T*_Land, Commit, 2015, Norm_ (°C), are marked orange in Fig. 1. Even with this normalisation, for many GCMs the mean equilibrium committed warming over land and for current GHG concentration levels remains higher than 1.5 °C.

**Figure 2:**
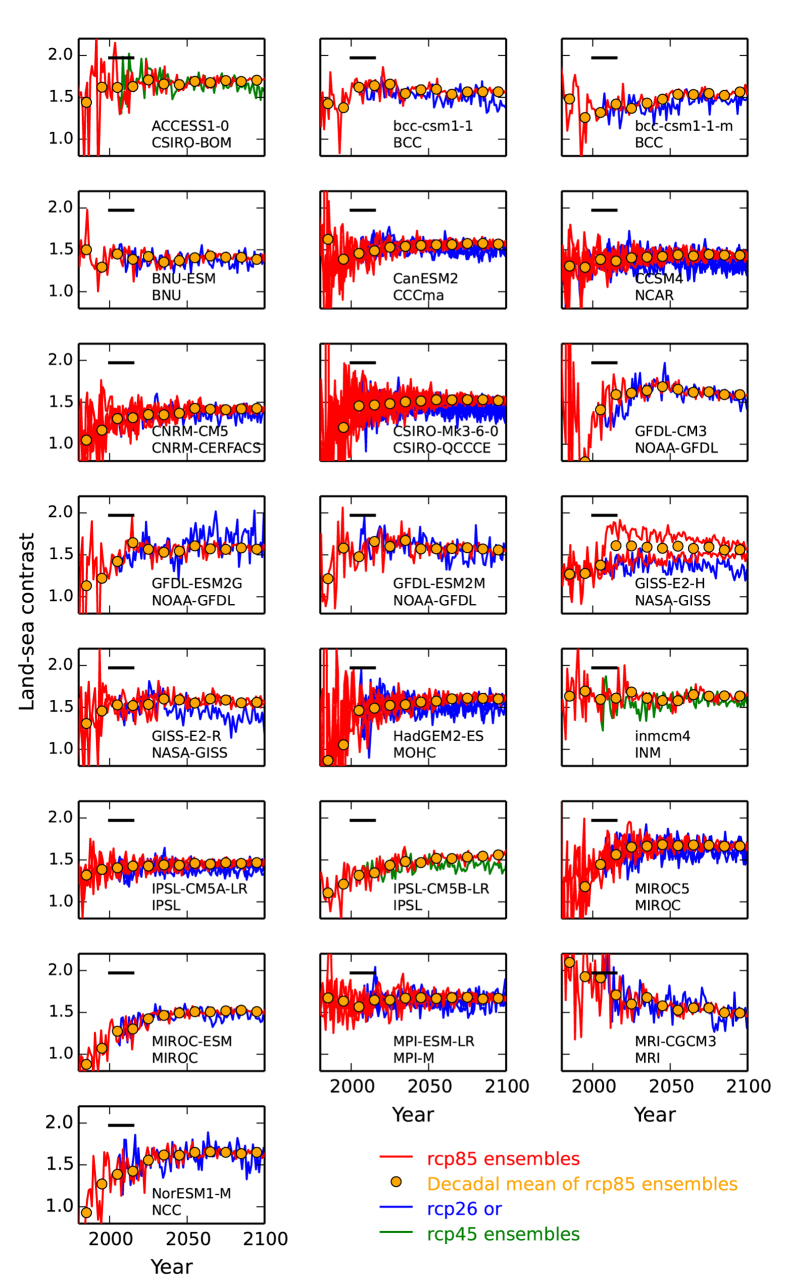
Land-sea warming contrast. For each GCM and associated research centre named in panels, for period 1980–2100, shown are model estimates of ensemble- and decadal-mean land-ocean temperature contrast, *v,* as orange dots. These are for the rcp85 scenario. Yearly calculations of *v* for rcp85 are given as red curves for each available model ensemble member. Also shown are ensemble members for rcp26, as blue curves. Where rcp26 simulations are unavailable, then rcp45 is given instead and as green curves.

Figure 2 provides additional information on the land-sea temperature contrast, presenting timeseries of *v* for each GCM. After approximately year 2010 this becomes relatively invariant for each model, and where this transition to a generally well-defined quantity at the beginning of the 21^st^ Century has been the subject of analysis^18^. Orange dots in Fig. 2 are decadal means of the rcp85 simulations (and ensemble means where more than one rcp85 simulation exists for any particular GCM). Individual rcp85 simulations are red curves. For each climate model, an overall *v* value is calculated as ensemble-mean for rcp85 scenario and between years 2010 and 2099 inclusive. These numbers are used in the equation above for calculating Δ*T*_Land, Commit, 2015_. The mean value of *v* across GCMs is 1.55 and with a low standard deviation of 0.084. To test our *v* values are appropriate to any discussion of stabilisation at 1.5 °C or 2.0 °C warming thresholds, additionally shown are timeseries of *v* for the models’ projections for low emission high mitigation scenario rcp26 (blue curves) or where this is unavailable, then for rcp45 (green curves). Most models project *ν* values to be similar between emissions scenarios. Marked in each panel of Fig. 2 as horizontal black line is mean *v* for period 2001–2015, for the NCDC/NESDIS/NOAA re-analysis observational dataset^16^. This value is higher than most models for that period.

Climate “pattern-scaling”^9^,^19^ capitalises on many features of projected alterations in monthly local meteorology being approximately linear in mean annual warming over land, Δ*T*_Land_. This includes local temperature changes, and allows interpolation away from the relatively small set of scenarios used to drive GCMs. Here, for each GCM *i* in Fig. 1, each spatial position over land *j* and each month *k*, a pattern value Δ*T*′_Land, Patt_ (*i*, *j*, *k*) (°C °C^−1^) is derived. This is the regression co-efficient of monthly and local temperature rise against global mean annual land warming Δ*T*_Land_(*i*) for each GCM. The scaling then allows estimation of local grid level monthly committed warming for contemporary atmospheric gas concentration, based on each GCM *i* emulated. Values are calculated by multiplying these patterns by Δ*T*_Land, Commit, 2015, Norm_ i.e. the orange values in Fig. 1.

**Figure 3:**
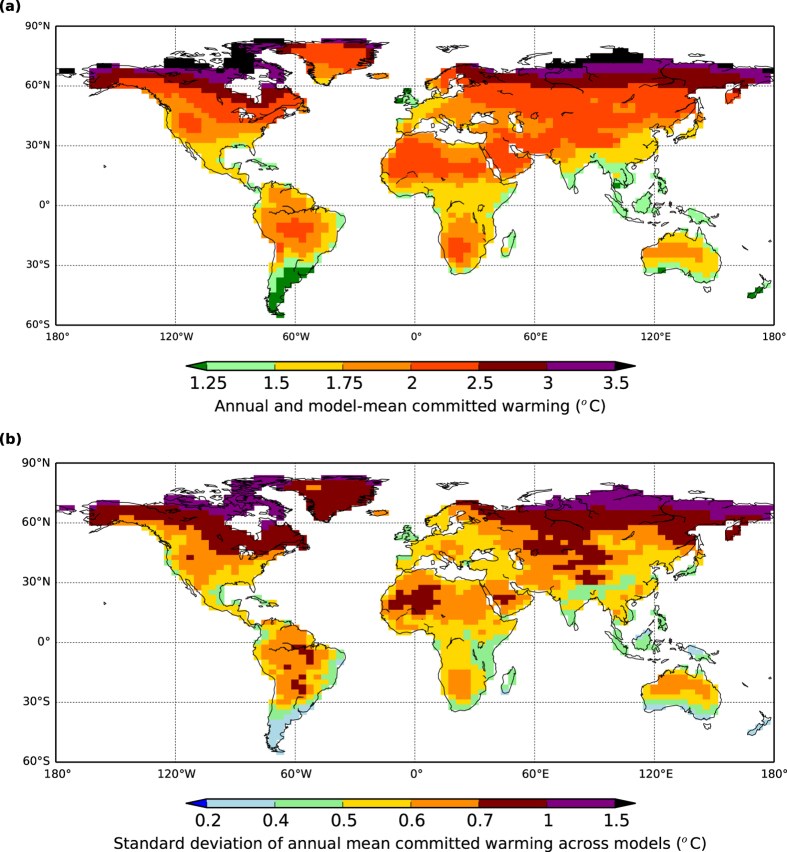
Annual mean GCM-based estimates of committed warming. Maps of calculated equilibrium warming levels for contemporary year 2015 greenhouse gas concentrations, based on knowledge of individual GCM climate sensitivity, land-ocean contrast and regional pattern-scaling. Panel (a) is multi-model mean of annual warming and panel (b) is the standard deviation between the GCMs emulated by scaling. GCMs considered are the same as those listed in Fig. 1. Figure created with Basemap module of python software package Matplotlib, version 1.3.1 (http://matplotlib.org/1.3.1).

In Fig. 3a, we average across GCMs *i* and months *k* of estimated local committed warming given by Δ*T*_Land, Commit, 2015, Norm_ (*i*) × Δ*T*′_Land, Patt_ (*i*, *j*, *k*) to get overall model-mean and annual-mean maps of estimated equilibrium warming. Model patterns are mapped on to a common grid of 2.5° latitude and 3.75° longitude. Figure 3 shows the model-mean estimates of committed annual warming over land are greater than 1.5 °C at most land points, and greater than 2.0 °C for many regions. Noticeable is that the places of least warming are generally nearer oceans. In addition, the highest warming levels are those in the Northern hemisphere and towards the pole. This strong latitudinal feature corresponds well to similar geographical patterns of emerging warming observed in the historical temperature records (ref. [20]; their zonal Fig. 3 showing high latitude present-day warmings already of order 1.2 °C since pre-industrial times). Figure 3b shows the standard deviations of the annual mean warmings for each GCM *i*, with the largest model spread of estimates in regions of highest model-mean warming.

**Figure 4:**
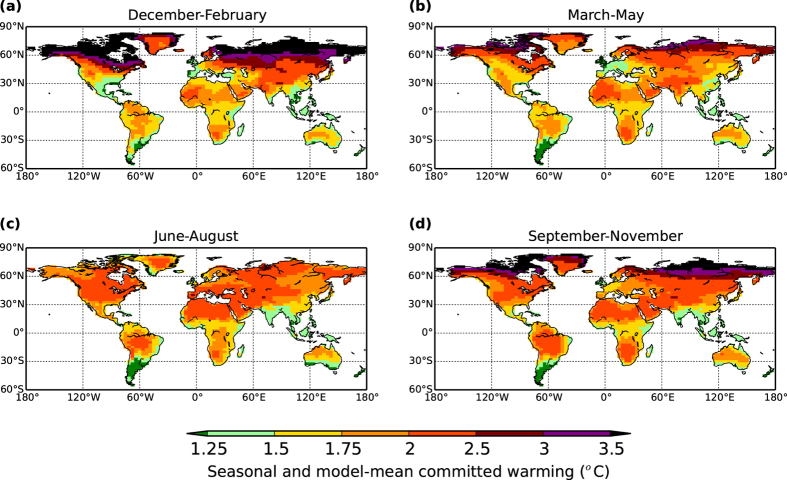
Seasonal mean GCM-based estimates of committed warming. As for Fig. 3a, but presented instead as seasonal means averaged across the GCMs emulated. Panels (**a–d**) represent the seasons as given in the panel titles. Figure created with Basemap module of python software package Matplotlib, version 1.3.1 (http://matplotlib.org/1.3.1).

In Fig. 4, we show the multi-model mean temperature rises, but now disaggregated in to average committed warmings for each season. Whilst there are strong similarities between seasons, and including less warming near major coastlines, notable is the very high warmings for northern latitudes during the boreal winter. This enhanced seasonal warming is a signal that again has also been noted as appearing in recent temperature measurements^21^, and including specifically for East Asia^22^.

## Discussion

There is strong evidence that even for current levels of atmospheric GHGs, there is a very high probability that the planet is committed to a mean warming over land greater than 1.5 °C relative to pre-industrial times. Such warming could be greater than 2.0 °C, and in particular for large continental regions away from coastlines. These projections remain, even when normalising to account for the observation that the past decade has seen warming rates lower than estimates by most climate models. Thermal inertia due to oceanic draw-down of heat implies that the current level of global warming is less than that of an equilibrium “committed” climatic state at contemporary GHG levels. Additionally, across all climate models, there is an especially robust and well-defined signal of much higher mean warming levels over land compared to the global mean.

Enhanced warming over land will affect terrestrial ecosystems, crop viability, glacier melt and cause other impacts^23^,^24^, along with human health implications of higher temperatures including within cities^25^. Increased and related leaf-level temperatures will raise vegetation respiration rates. The amount to which this will offset vegetation fertilisation due to higher CO_2_ concentrations will influence the global carbon cycle^26^, and therefore also affect “permissible” emissions to constrain warming to any prescribed limit. Raised warming could also modulate complex circulation patterns^27^, adjusting the local hydrological cycle with implications for rainfall patterns. Detailed analysis over the coming years of high-resolution regionally-based GCM outputs will link different proposed global temperature limits to the more local features of higher land warmings and other meteorological changes. This will aid understanding of likely adaptation needed to deal with any adverse impacts at different global warming levels.

The IPCC has called for a special report in year 2018 to determine what climate impacts can be expected for 1.5 °C of global mean warming. It has been argued^28^ that date might be too soon to understand in full any differences between such a warming level, and the alternative of 2.0 °C. Demonstrated here is if heavy emissions reductions prevent either value being crossed, the robust inter-model estimates of land-sea warming contrast implies significantly higher temperatures will still be experienced over land. This must be taken in to account in any debate of 1.5 °C versus 2.0 °C as a global mean threshold aspiration. Unfortunately determining the equilibrium GHG concentrations compatible with any prescribed warming levels, either globally or over land, remains difficult. This is due to large model differences in estimates of planetary climate sensitivity.

## Additional Information

**How to cite this article**: Huntingford, C. and Mercado, L. M. High chance that current atmospheric greenhouse concentrations commit to warmings greater than 1.5 °C over land. *Sci. Rep.* **6**, 30294; doi: 10.1038/srep30294 (2016).
